# Highly efficient reusable superhydrophobic sponge prepared by a facile, simple and cost effective biomimetic bonding method for oil absorption

**DOI:** 10.1038/s41598-021-91396-9

**Published:** 2021-06-07

**Authors:** Jiaqi Wang, Yan Chen, Qinyao Xu, Miaomiao Cai, Qian Shi, Junkai Gao

**Affiliations:** 1 School of Naval Architecture and Maritime, Zhejiang Ocean University, Zhoushan, 316022 China; 2 School of Marine Engineering Equipment, Zhejiang Ocean University, Zhoushan, 316022 China

**Keywords:** Pollution remediation, Composites

## Abstract

Superhydrophobic sponges have considerable potential for oil/water separation. Most of the methods used for superhydrophobic modification of sponges require toxic or harmful solvents, which have the drawbacks of hazardous to environment, expensive, and complex to utilize. Moreover, the hydrophobic layer on the surface of sponge is often easily destroyed. In this paper, a highly efficient superhydrophobic sponge with excellent reusability was developed by using a facile, simple and environmentally friendly dopamine biomimetic bonding method. Different types of sponges, such as melamine, polyethylene or polyurethane sponge wastes, were used as raw materials to prepare superhydrophobic sponges, which possess the advantages of inexpensive and abundant. The effects of different dopamine polymerization time and different hydrophobic agent dosage on the hydrophobicity and oil absorption capacity of melamine sponges were optimized. The study results showed that the water contact angle of the superhydrophobic sponge could reach 153° with excellent organic solvent absorption capacity of 165.9 g/g. Furthermore, the superhydrophobic sponge retained approximately 92.1% of its initial absorption capacity after 35 reutilization cycles. More importantly, the dopamine biomimetic bonding superhydrophobic modification method can be used for different types of sponges. Therefore, a universally applicable, facile, simple and environmentally friendly superhydrophobic modification method for sponges was developed.

## Introduction

Oil/water separation and oil absorption are hot researched topics since the discharge of industrial oily wastewater and the occurrence of oil spill accidents have resulted in environmental contamination in recent years^1–3^. To minimize the impacts of oil pollution, an ongoing challenge facing researchers is developing materials with low cost and high efficiency for oil absorption. Methods of oil spill treatment include dispersants, booms, skimmers, sorbents, and bioremediation^4–8^. Among these methods, absorption has the advantages of high efficiency, easy operation, and high absorbability.

Absorptive materials include nanoparticles, two-dimensional (2D) materials and three-dimensional (3D) materials. Oleophilic nanoparticles with great absorbability, such as zeolite, activated carbon and silica, have been investigated for the removal of oil^9–11^. 2D materials with excellent oil–water separation ability include synthetic films, meshes, and fabrics^12–14^. Hydrophobic porous 3D materials and their outstanding absorption performance towards oils and organic solvents have investigated recently^15^. 3D hydrophobic materials, which have a large storage capacity, can absorb and desorb oil immediately. However, the synthesis of materials, such as carbon nanotube (CNT) foam, rubber/graphene composite materials, cellulose aerogels and graphene aerogels, faces the challenges of complicated operation, high cost and causing environmental pollution^16,17^. Among these 3D materials, sponges not only possess the advantages of porous structure, great flexibility, and commercial availability^18^ but can also be chemically modified^19^.

Sponges are commonly applied for sound absorbance, heat preservation and fire prevention^20^, and the increased consumption of sponges leads to the production of many sponge wastes, which cannot be degraded naturally. Therefore, sponge wastes, which are inexpensive and abundant, are a promising material for oil spill absorption. More importantly, the reutilization of sponge wastes is very beneficial for the protection of the environment. However, when sponges are directly applied for spilled oil recovery, they also absorb large amounts of water due to the amphiphilicity of the sponge surface. This amphiphilicity leads to the sponge having difficulty of selectively absorbing oil from the water, which limits the practical application of these sponges^21^.

To overcome this challenge, recent studies have focused on the surface modification of sponges, which could alter the hydrophilic surface to make it hydrophobic. A hydrophobic surface would provide the sponge with good water repellency during the separation process of oil absorption^22–24^. However, most of the hydrophobic layer is easily destroyed and desorbed due to the weak adhesion between the superhydrophobic coating and the sponge skeleton. For the purpose of enhancing the stability of the hydrophobic layer, it is important to fabricate a robust superhydrophobic coating on the surface of the sponge^25^.

An effective strategy to overcome the above challenge is using additional adhesives to connect the sponge and the hydrophobic agents^19,26^. However, traditional adhesives, such as silane agents, fluorides, and epoxy resin, have the drawbacks of harmfulness to the environment, high cost, and complex preparation processes. In addition, large amount of adhesives can bind plenty of hydrophobic materials and block the channels of the sponge, which results in a decrease in the oil absorption capacity of the sponge^25,27^. Therefore, it is imperative to develop a facile, environmentally friendly and economical method for fabricating hydrophobic sponges.

Dopamine can self-polymerize under weak alkaline conditions to form a polydopamine (PDA) layer, which can be deposited on numerous material surfaces^28,29^. Moreover, the PDA particles can act as a secondary reaction platform while still retaining its long-lasting adhesion, which is attributed to the reactive functional groups of catechol hydroxyl and amino groups on the surface of PDA. Furthermore, dopamine biomimetic modification has many advantages, including nontoxicity, mild conditions and easy operation. Taking advantage of the excellent adhesion of PDA, in this study, the catechol compounds of PDA were used to combine hydrophobic molecules and sponge skeletons as bioadhesive to ensure strong binding of the hydrophobic materials on melamine sponge (MS)^30,31^.

**Figure 1:**
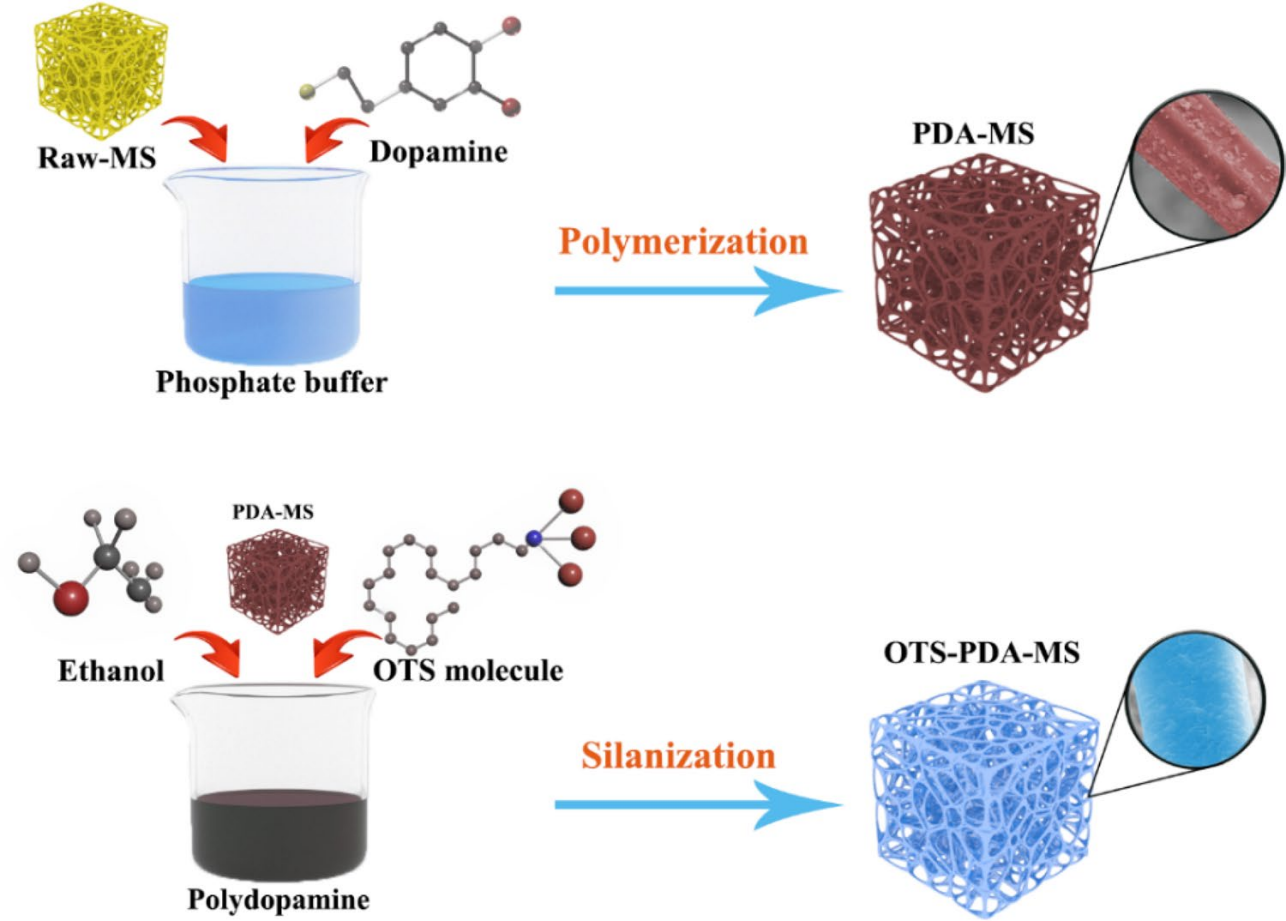
Schematic illustration of the fabrication of OTS-PDA-MS.

In this work, inspired by the prominent adhesive ability of PDA, the hydrophobic OTS was applied for modification different types of sponges via PDA as a binder and a highly efficient superhydrophobic sponge (OTS-PDA-MS) was prepared by using a facile, eco-friendly and cost-effective dopamine biomimetic bonding method. The influences of dopamine with different polymerization time for binding the hydrophobic OTS on MS were first deeply studied. And moreover, the different hydrophobic agent dosage on the hydrophobicity and oil absorption capacity of melamine sponges were optimized. A schematic illustration of the simple preparation process of OTS-PDA-MS is illustrated in Fig. 1. The results demonstrated that dopamine biomimetic bonding was a highly efficient strategy to prepare superhydrophobic sponges for oil absorption.

## Experimental

### Materials

MS was supplied by Yiwu, Zhejiang Sponge Products Co., Ltd. The polyethylene sponge and polyurethane sponge were obtained from Yijia Sponge Products Co., Ltd. Dopamine hydrochloride and trichloro(octadecyl)silane (OTS) were purchased from Sigma Aldrich. Anhydrous ethanol, sodium phosphate dibasic anhydrous and sodium phosphate monobasic anhydrous were provided by Sinopharm Chemical Reagents Co., Ltd. Sudan III was purchased from Shanghai Mai Kun Co., Ltd. All chemicals were used as received.

### Preparation of superhydrophobic sponge

The superhydrophobic sponge was prepared by using a biomimetic adhesion strategy. Specifically, dopamine hydrochloride (0.25 g) was added into phosphate buffer solution (sodium phosphate dibasic anhydrous of 0.072 g and sodium phosphate monobasic anhydrous of 1.69 g in 50 mL distilled water) with a pH of 8.5 and stirred for 24 h to form a PDA suspension. Then, MS was immersed into the PDA suspension and stirred for 24 h. One milliliter of OTS was mixed with 40 mL of ethanol (1:40) and stirred for 10 min, and the above solution was added to the MS and PDA mixture and stirred for 2 h. Then, the mixture was treated by ultrasound sonication for 10 min. Finally, the sponge was rinsed with distilled water and ethanol, and the excess solution was removed from the sponges by direct squeezing, which prevented OTS from concentrating on the surface of the sponge. Finally, hydrophobic MS was obtained after drying at 45 °C for 12 h. For comparison, the melamine sponge modified directly by OTS (OTS-MS) was prepared without dopamine bonding by using the same synthesis procedure as for OTS-PDA-MS, and the polyethylene sponge (PES) and polyurethane sponge (PUS) were also modified by OTS based on PDA biomimetic bonding to prepare OTS-PDA-PES and OTS-PDA-PUS, respectively, using the same synthesis procedure as for OTS-PDA-MS.

### Oil absorption capacity test

First, the oil or organic solvent was sufficiently absorbed by the sponge. Afterwards, each piece of sponge was weighed, and the oil absorption quantity (q) of OTS-PDA-MS was calculated by the following formula^32^:

$$ q = \left( {M_{1} - M_{2} } \right)/M_{2}$$

where *M*_2_ and *M*_1_ are the weights of the sponge before and after absorption of oil. All absorption experiments were performed 5 times, and the average absorption value was reported.

### Oil recyclability test

To test the recyclability of the superhydrophobic sponge, a simple absorption–desorption process was utilized. First, the superhydrophobic sponge was immersed in lubricating oils for 5 min to ensure the desorption saturation of the superhydrophobic sponge for lubricating oils. Then, the saturated superhydrophobic sponge was centrifuged to remove the oils. The desorption efficiency (r) of the superhydrophobic sponge was calculated according to the following equation:

$$ r = 1 - \left( {m_{1} /m_{0} } \right) \times 100\%$$

where *m*_0_ represents the weight of the saturated superhydrophobic sponge after the sorption of oil and *m*_1_ represents the weight of the superhydrophobic sponge after centrifugation. All recycling experiments were performed three times, and the average experimental value was reported.

### Density and porosity

Apparent densities were calculated by measuring their masses and dimensions. The porosity was calculated using the following equations^33^:

$$ Porosity = \left( {1 - \rho /\rho_{s} } \right) \times 100\%$$

where *ρ* is the bulk density of the sponges and *ρ*_*s*_ is the raw material density of the sponges.

### Anti-oil fouling analysis

The anti-oil fouling property of the OTS-PDA-MS was evaluated by two indexes of the flux recovery ratio (FRR) and irreversible fouling ratio (RIR), which were calculated by the formula^34^:

$$FRR=\left(\frac{{J}_{a,2}}{{J}_{a,1}}\right)\times 100\%$$

$$RIR=\left(\frac{{J}_{a,1}-{J}_{a,2}}{{J}_{a,1}}\right)\times 100\%$$

where J_a,1_ is original absorption capacity and J_a,2_ is last absorption capacity after desorption. In general, FRR means that the variation of absorption capacity of sponges before and after use. The RIR indicate the changing rate of absorption capacity of sponges.

### Characterization

Contact angle measurements were carried out using a JY-82 Contact Angle Apparatus (Chengde Dingsheng Company, Ltd., Hebei) at ambient temperature. The characteristic functional groups of the modified sponge were determined by reflection-mode Fourier transform infrared spectroscopy (ATR-FTIR, Bruker, VERTEX70, Germany). Scanning electron microscopy (SEM, FEG-250, FEI, USA) was used to visualize the morphology of the sponges. The surface chemical compositions of the modified sponge were measured by X-ray photoelectron spectroscopy (XPS, ESCALAB 250Xi K-Alpha, Thermo Fisher). The compressive stress–strain tests were performed via (Shimadzu AG–X plus testing system).

## Results and discussion

### Surface morphological analysis

**Figure 2:**
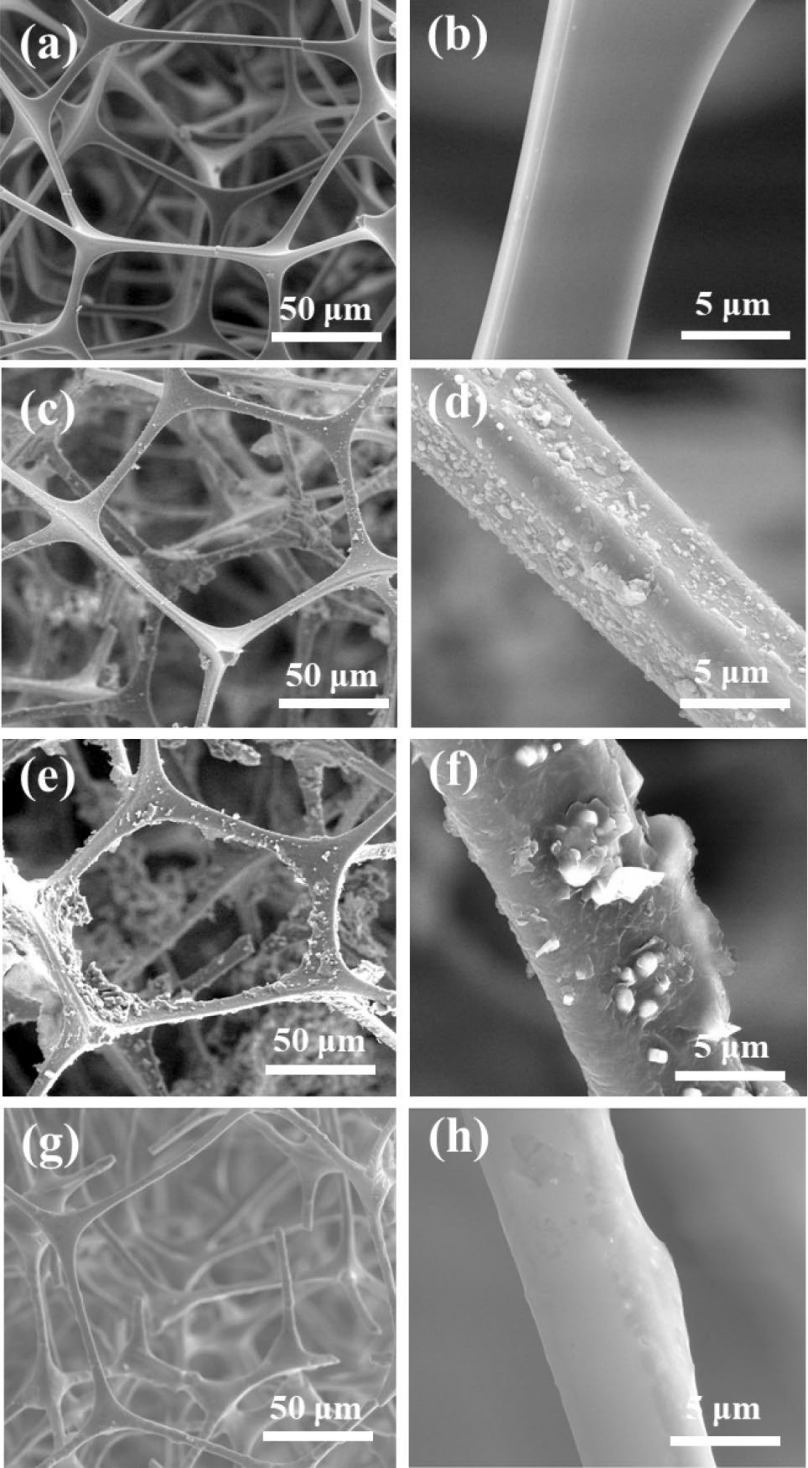
SEM images of sponges employed in this work. (**a**,**b**) Raw MS, (**c**,**d**) PDA-MS, (**e**,**f**) OTS-PDA-MS, (**g**,**h**) OTS-MS.

The SEM images of the MS before and after modification are shown in Fig. 2. As shown in Fig. 2a,b, the raw-MS possessed a distinct structure with a stable 3D-interconnected network and a highly porous structure with a size of 10–100 μm, which played an important role in the absorption and retention of oil. Additionally, the SEM images revealed that the raw sponge possessed a smooth skeleton surface.

The SEM images of PDA-MS (Fig. 2c,d) show that the skeleton of MS was coated with PDA. Since the PDA particles aggregated with each other, some nanoparticles formed a thin granular layer, and some assembled in agglomerates. Moreover, the PDA particles were formed on the surface of the sponge without damaging the intrinsic structure of MS. The countless microscale PDA particles were located on the skeleton, which resulted in the formation of a rough surface of PDA-MS, thereby improving the hydrophobicity of the sponges after OTS modification^35^.

After the PDA-MS was modified with OTS (Fig. 2e,f), the network structure of the sponge remained intact and excellent, which indicated the feasibility of the modification conditions. On the surface of the MS, a thin and smooth layer covered the PDA nanoparticle agglomerates. The results confirmed that the sponge was successfully coated by OTS through dip-coating, which is crucial for the preparation of superhydrophobic materials. As shown in Fig. 2g,h, the layer on the surface of OTS-MS appeared smoother than the layer on OTS-PDA-MS and the surface roughness of OTS-PDA-MS was higher than that of OTS-MS, which improved the hydrophobicity of OTS-PDA-MS.

### Chemical property analysis of MS, PDA-MS and OTS-PDA-MS

**Figure 3:**
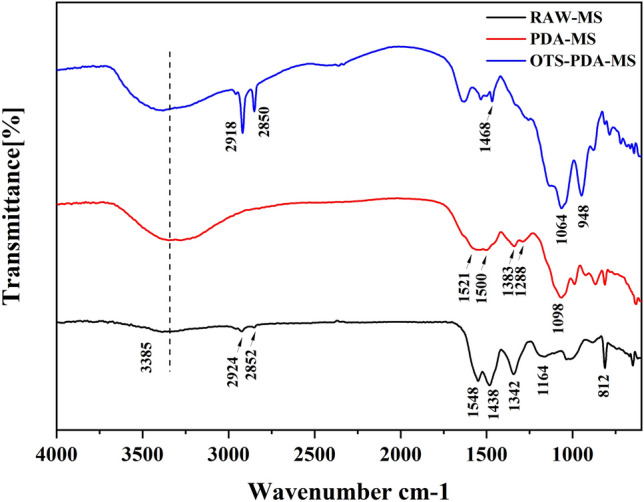
FTIR spectra of raw MS, PDA-MS and OTS-PDA-MS.

To determine the functional groups on the sponge surface, FT-IR analysis was conducted to detect the chemical components of raw MS, PDA-MS and OTS-PDA-MS (Fig. 3). The spectrum of the untreated MS displayed a blunt peak at 3385 cm^-1^, which can be assigned to N–H (the secondary amine) stretching^36^. With the exception of N–H stretching, the newly formed bands at 3100–3400 cm^-1^ are attributed to O–H stretching vibrations and correspond to the hydroxyl group of PDA-MS. In the spectrum of OTS-PDA-MS, the peak intensity of O–H was slightly weaker than that of PDA-MS because the O–H group of PDA-MS was replaced by hydrophobic groups in the molecules of OTS. Moreover, the two small peaks in the spectrum of OTS-PDA-MS at 2918 and 2850 cm^-1^ can be attributed to –C–H stretching asymmetrical stretching vibrations and symmetrical stretching vibrations, respectively^37^ (called Fermi vibration). These double peaks in the spectrum of OTS-PDA-MS were more intense than those of PDA-MS and raw MS, and they were caused by the hydrophobic groups of alkylsilane in OTS. In the spectrum of PDA-MS, the double absorption bands at 1500 cm^-1^ and 1521 cm^-1^ can be attributed to overlapping of the C=C resonance vibration in aromatic rings. In addition, PDA-MS displays a specific peak at 1098 cm^-1^ ascribed to the C–O of PDA^38–40^, and the peaks at 1383 cm^-1^ and 1288 cm^-1^ can be attributed to phenolic C–O–H bending and stretching vibrations^41^. In addition, in the spectrum of OTS-PDA-MS, the intensity of the peak at 948 cm^-1^ significantly increased due to the overlapping of the C–H bending peak, and a new Si–O–Si stretching peak (1064 cm^-1^) evolved due to the formation of silane on the surface of the skeleton^42^. The presence of these groups indicate that the sponge was successfully modified with the OTS.

**Figure 4:**
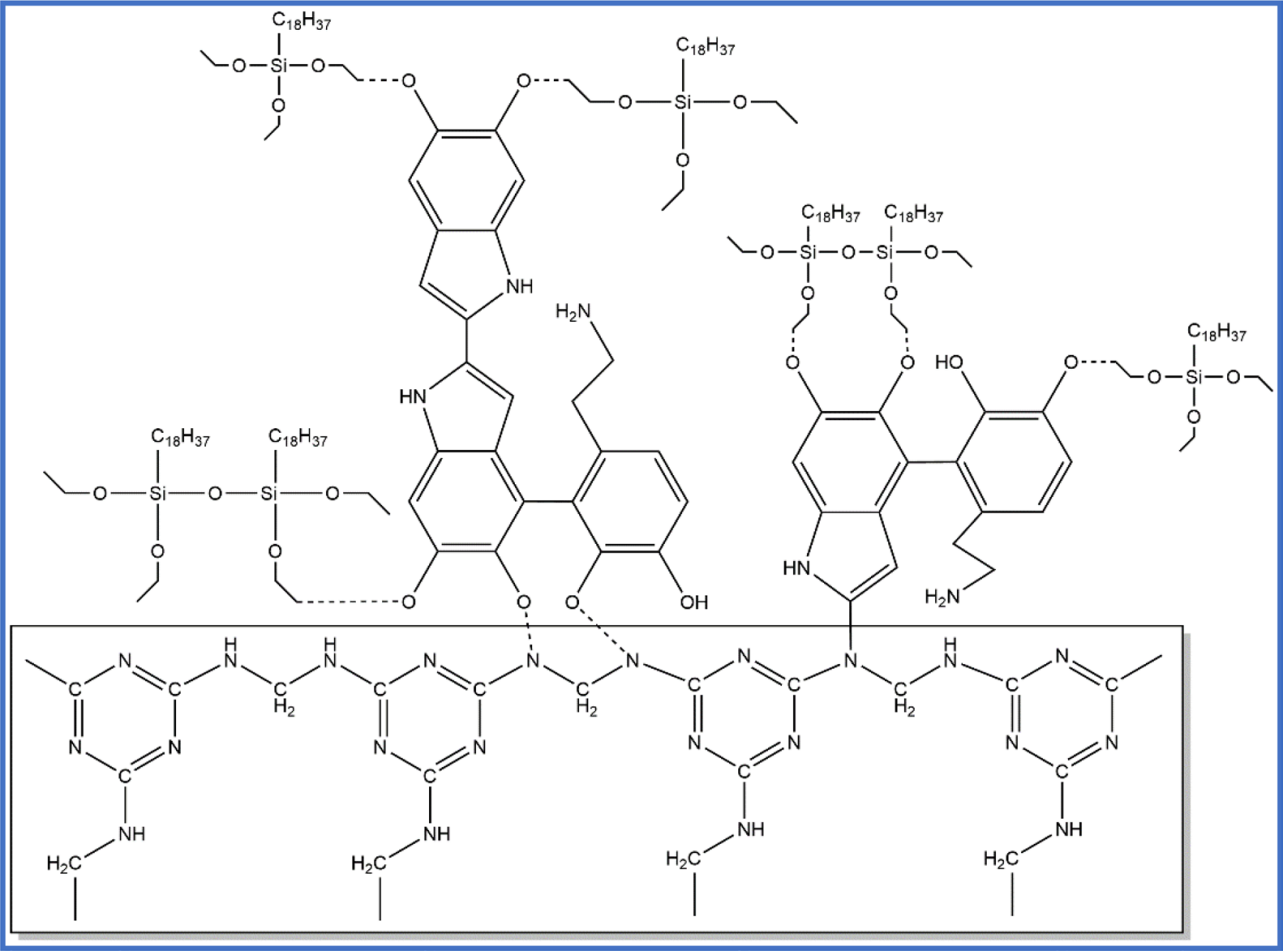
Interaction mechanism between PDA and OTS on the surface of MS.

Figure 4 illustrates the interaction mechanism between PDA and OTS on the surface of MS. The octadecyltriethoxysilane interacted with PDA through hydrogen bonds between the –OH groups in PDA and the –OH groups in octadecyltriethoxysilane^43–45^.

The PDA interacts with the MS through hydrogen bonds between the -OH groups and the N–H groups and covalent bonds between the C–H groups and the N–H groups. And OTS was added to an ethanol solution and reacted with ethanol to form a hydrophobic material of octadecyltriethoxysilane, then crosslinked with PDA and attached to the surface of the sponge. The PDA molecules can connect to octadecyltriethoxysilane through covalent bonds between the C–H groups in PDA and the –OH groups in octadecyltriethoxysilane. Each silane molecule in octadecyltriethoxysilane can connect with neighboring silane molecules to form siloxane bonds and then form a network of Si–O–Si bridges. Moreover, these siloxane bonds and Si–O–Si bridges anchored to the surface of PDA exhibit hydrogen bonds and covalent bonds^46–50^.

### Chemical composition analysis of MS, PDA-MS and OTS-PDA-MS

**Figure 5:**
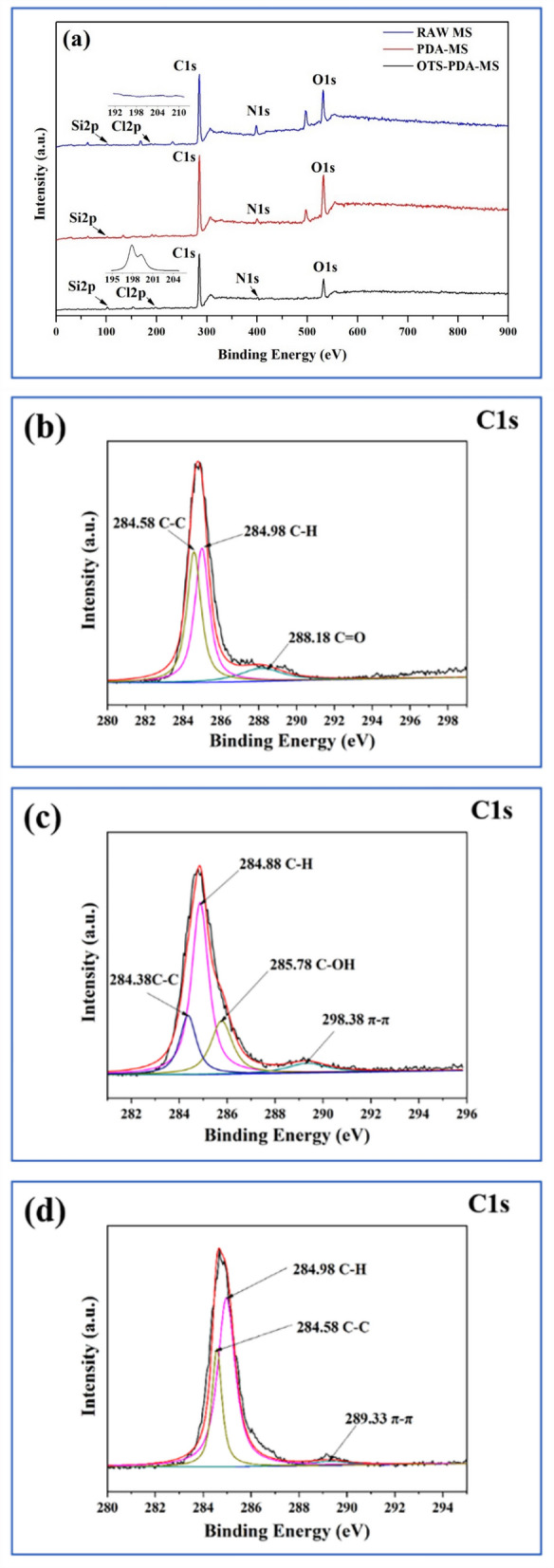
(**a**) XPS survey spectra of the raw MS, PDA-MS and OTS-PDA-MS; (**b**–**d**) are the C1*s* XPS spectra of raw-MS, PDA-MS and OTS-PDA-MS, respectively.

To further confirm that PDA and OTS were successfully loaded on the surface of the sponge skeletons, XPS analysis was performed to investigate the surface chemical composition of the sponge before and after modification. As shown in Fig. 5a, C1*s* and O1*s* were the main elements. For raw MS, a strong C1*s* peak and the other two characteristic peaks of nitrogen (N1*s*) and oxygen (O1*s*) were observed at 284.88 eV, 399.88 eV, 531.33 eV, and the peak at 101.88 eV could be attributed to Si2*p*^42^. After the MS was grafted with PDA and OTS, N1*s* and C1*s* had subtle differences in the binding energies of the element peaks. Compared with pristine MS, which was not detected with Cl2*p*, OTS-PDA-MS had a binding energy of the Cl2*p* peak at 198.08 eV^43^. Cl elements occurred because of alkylsilane derived from OTS, which was show at small black peak 195–204 eV. As shown in Fig. 5c, compared with the raw MS without the peak of C–OH (Fig. 5b), the peak of C–OH at 285.78 eV represented the oxygen in the molecules of PDA, which indicated that PDA was successfully introduced on the surface of the sponge skeleton. The signal intensity of oxygen increased, which reflects the increased O/N atom ratio of raw MS to PDA-MS. Comparing raw MS and PDA-MS, the O/N atom ratios were 2.81 and 6.55, respectively, which is consistent with the large increase in the hydroxyl band intensity in the infrared spectrum of PDA-MS compared to raw MS. After hydrophobic modification with OTS, in the spectrum of OTS-PDA-MS, the C1*s*, O1*s* and N1*s* peak intensities notably decreased. In contrast, the Si2*p* peak intensity in the spectrum of OTS-PDA-MS increased after OTS modification. Compared to the spectrum of PDA-MS (Fig. 5c), in the high-resolution XPS data of C1*s* of OTS-PDA-MS (Fig. 5d), the peak of C–OH was not observed, indicating that most of the -OH groups were replaced by OTS molecules^19^.

### Wetting behavior of different MS

**Figure 6:**
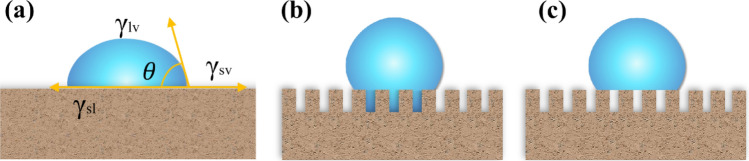
Schematic illustration of typical wetting behaviors of a droplet on a solid surface with and without roughness. (**a**) Young’s model, (**b**) Wenzel’s model, and (**c**) Cassie’s model.

As shown in Fig. 6a, the contact angle of a liquid droplet is a parameter for describing the surface wetting behavior of materials. The degree of wettability of liquid droplets on a solid surface is commonly described in terms of contact angle (CA) $\theta$ which can be expressed by Young's Eq.^51^:

$$cos\theta =\frac{{\gamma }_{sv}-{\gamma }_{sl}}{{\gamma }_{lv}}$$

where ${\gamma }_{sv}$, ${\gamma }_{sl}$ and ${\gamma }_{lv}$ are the interfacial tensions between solid and vapour, solid and liquid, liquid and vapor, respectively. Depending on the value of WCA, the surface properties are categorized as hydrophilic ($\theta <90^\circ$), hydrophobic ($90<\theta <150^\circ$) and superhydrophobic ($\theta >150^\circ$).

As shown in Fig. 6b, for a liquid droplet on surface of solid with high roughness, the droplet will contact with a part of the rough surface and the gaps between the roughened surface of the solid are filled by liquid droplet, which could describe in the Wenzel’s model^52^:

$$cos{\theta }^{*}=Rcos\theta $$

where *R* signifies the roughness factor and $\theta$ are the CA of a droplet on a flat solid surface. Since the value of *R* is always larger than 1, the introduction of *R* will enhance the wettability of a flat solid surface. If the flat solid surface has a CA greater than 90°, the rough surface could further increase CA of solid. However, if it has a CA less than 90°, the surface roughness would decrease the CA of solid adversely^51^.

As shown in Fig. 6c, the droplet can suspend on the top of the projections. However, for extremely high roughness, assuming a 180° liquid contact angle for air, the trapped air-pockets under water droplets are considered as the superhydrophobic medium, which could repel water penetration. The liquid contact angle for air can be expressed the Cassie’s model^27^:

$$cos{\theta }^{*}=fcos\theta +\left(1-f\right)cos180^\circ =fcos\theta +f-1$$

where θ* and *θ* are apparent contact angle and contact angle on ideal flat surface. *f* is the surface fraction of solid.

The wetting behavior of sponges was tested via both of the above theories. The static contact angle of superhydrophobic surface was greater than 150° and slide angle was lower than 10°.

**Figure 7:**
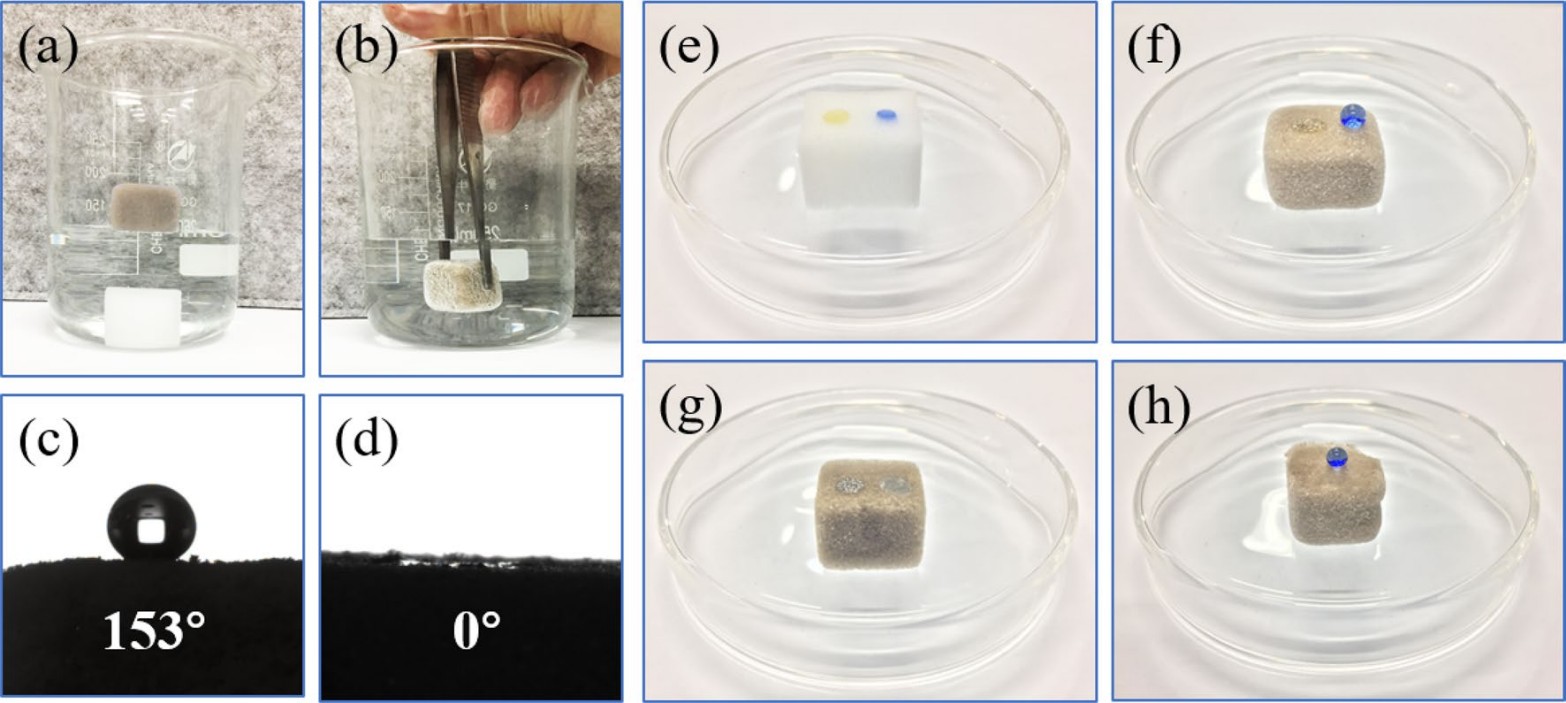
(**a**) Floating experiments of raw MS and OTS-PDA-MS. (**b**) Immersion experiment of OTS-PDA-MS. (**c**, **d**) WCA and oil CA test of OTS-PDA-MS. Photographs of oil and water droplets on (**e**) raw MS, (**f**) PDA-MS and (**g**, **h**) OTS-PDA-MS.

The functionalized sponges were evaluated with water contact angle (WCA) experiments, which were performed to determine the wettability of the modified sponges. Figure 7a shows a photograph of the modified MS floating on the water surface due to its water repellency, while the raw MS sinks beneath the water surface. When immersing the modified sponge by means of an external force, a silver mirror-like reflection appeared on the surface of the sponge, as shown in Fig. 7b, because air bubbles were entrapped between the modified sponge and the surrounding water.

As shown in Fig. 7e, g, pristine MS and PDA-MS exhibited almost no repellency to water (dyed with blue ink), which was attributed to the existence of abundant hydroxyl groups and the 3D-interconnected structure of the raw sponge for liquid absorption. However, after hydrophobic functionalization, the rough surface of MS and the hydrophobic groups resulted in a superhydrophobic surface. As shown in Fig. 7c, d, f OTS-PDA-MS showed excellent hydrophobicity with a water contact angle of 153°, while the oil droplet directly permeated through the interior of OTS-PDA-MS, leaving a minor puddle of oil on the surface. Additionally, lubricating oil droplets could completely penetrate the surface of OTS-PDA-MS in less than 5 s with a contact angle of almost 0° (see details in Video S1). Moreover, for the purpose of verifying the uniformity of the hydrophobic modification, the sponge was stripped from the exterior surface, and the internal structure of the sponge retained its repellency to water, as shown in Fig. 7h. This phenomenon revealed that the hydrophobic groups were deposited over the entire sponge skeleton.

**Figure 8:**
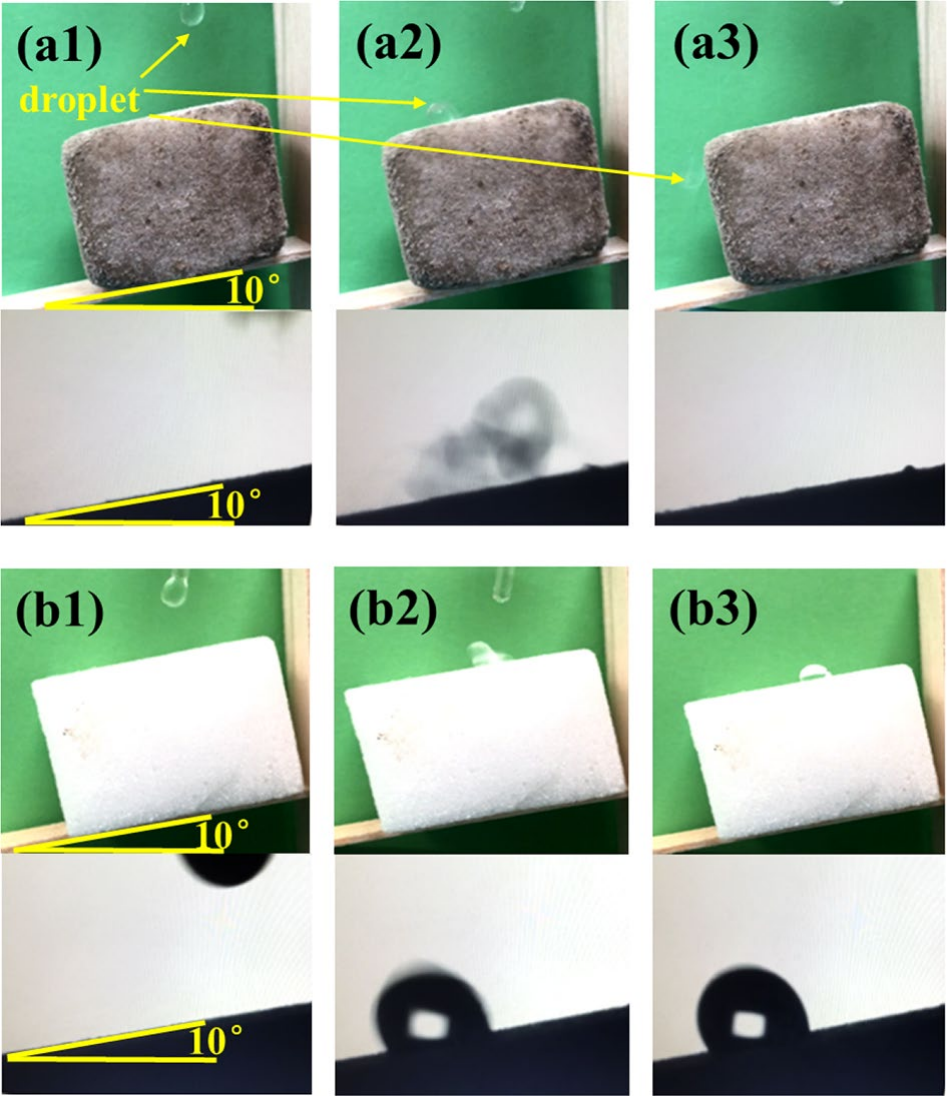
(**a1**–**a3**) The sliding angle test of OTS-PDA-MS. (**b1**–**b3**) The sliding angle test of OTS-MS.

To further studied the superhydrophobic surface of OTS-PDA-MS, the dynamic water contact angle was showed in Fig. 8a1–a3. The sliding angle (SA) of OTS-PDA-MS was only about 10° and the droplet on surface of OTS-PDA-MS was immediately slide away, which indicated excellent superhydrophobic surface of OTS-PDA-MS. However, as shown Fig. 8b1–b3, in the droplet on surface of OTS-MS was stuck, reflecting the hydrophobic surface of OTS-MS.

**Figure 9:**
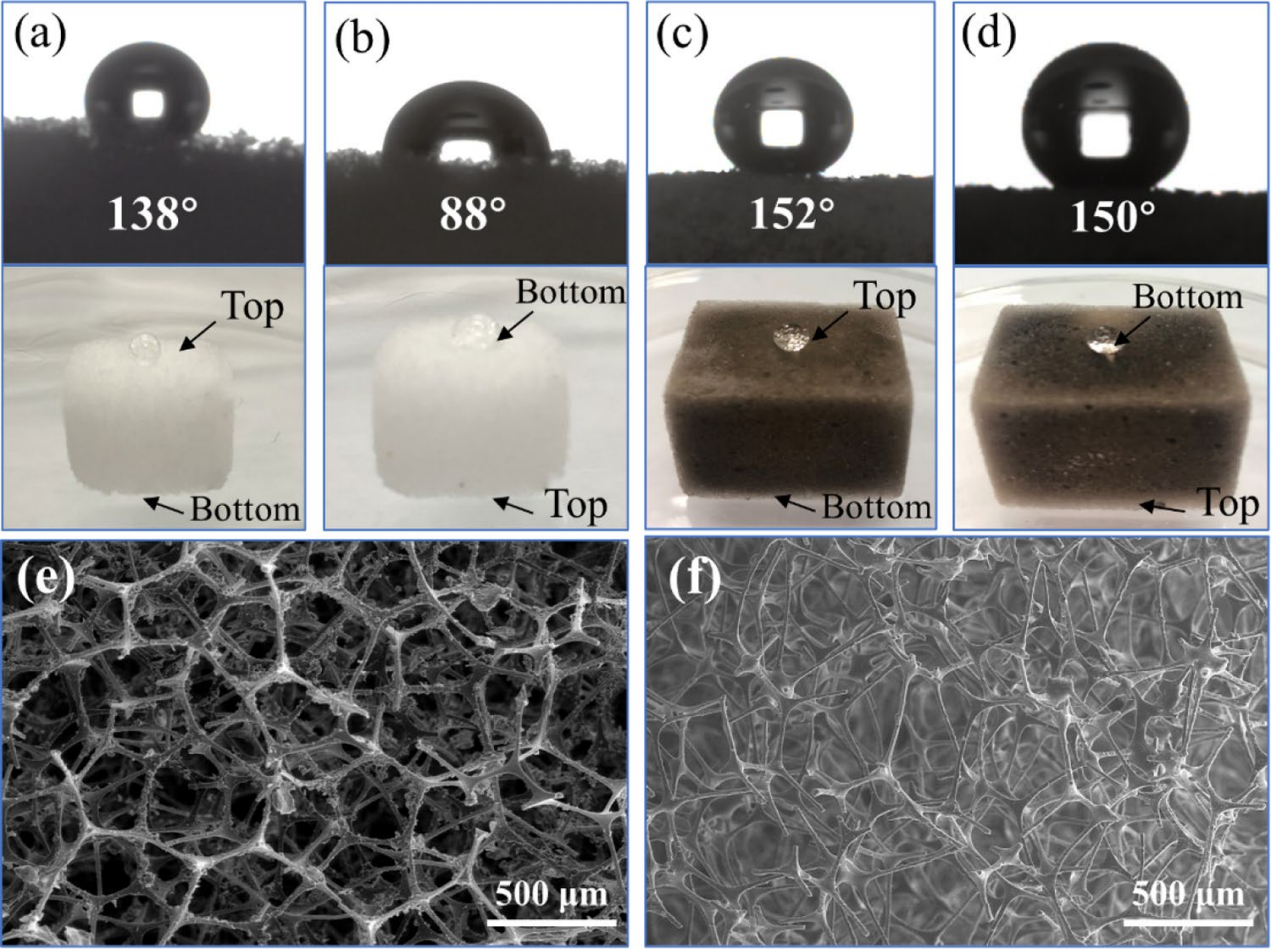
(**a**) WCA at the top of OTS-MS; (**b**) The WCA at the bottom of OTS-MS; (**c**) The WCA at the top of OTS-PDA-MS; (**d**) The WCA at the bottom of the OTS-PDA-MS; (**e**) the SEM image of OTS-PDA-MS; (**f**) the SEM image of OTS-MS.

To study the effect of PDA as a binder agent between MS and hydrophobic OTS, the MS was directly modified by OTS through hydrolytic processes. As displayed in Video S2, a drop of water was placed on the surface of OTS-MS and OTS-PDA-MS and monitored for 25 min. After 25 min, the droplet on the surface of OTS-PDA-MS was mostly unchanged, but the droplet on the surface of OTS-MS gradually permeated the interior of OTS-MS. Figure 9a,b showed the WCAs on different spots on the surface of OTS-MS, which were 138° at the top and 88° at the bottom of OTS-MS, indicating that the bottom part of OTS-MS was hydrophilic. Figure 9c,d showed the WCAs on different spots on the surface of OTS-PDA-MS, which were 152° at the top and 150°at the bottom of OTS-PDA-MS. The static contact angle has to greater than 150°, indicating that the superhydrophobic surface of OTS-PDA-MS. Furthermore, as shown in Fig. 9e,f, the surface skeleton of OTS-PDA-MS was covered by PDA particles and the OTS-MS appeared smooth surface. This phenomenon might be attributed to the nonuniformity of the hydrophobic layer on the surface of OTS-MS. In conclusion, PDA could be used as a strong binding agent between OTS and the sponge skeleton. Moreover, the use of PDA facilitated the uniform distribution of OTS on the surface of OTS-PDA-MS and reached superhydrophobic surface of OTS-PDA-MS.

### Effect of different polymerization time of PDA

**Figure 10:**
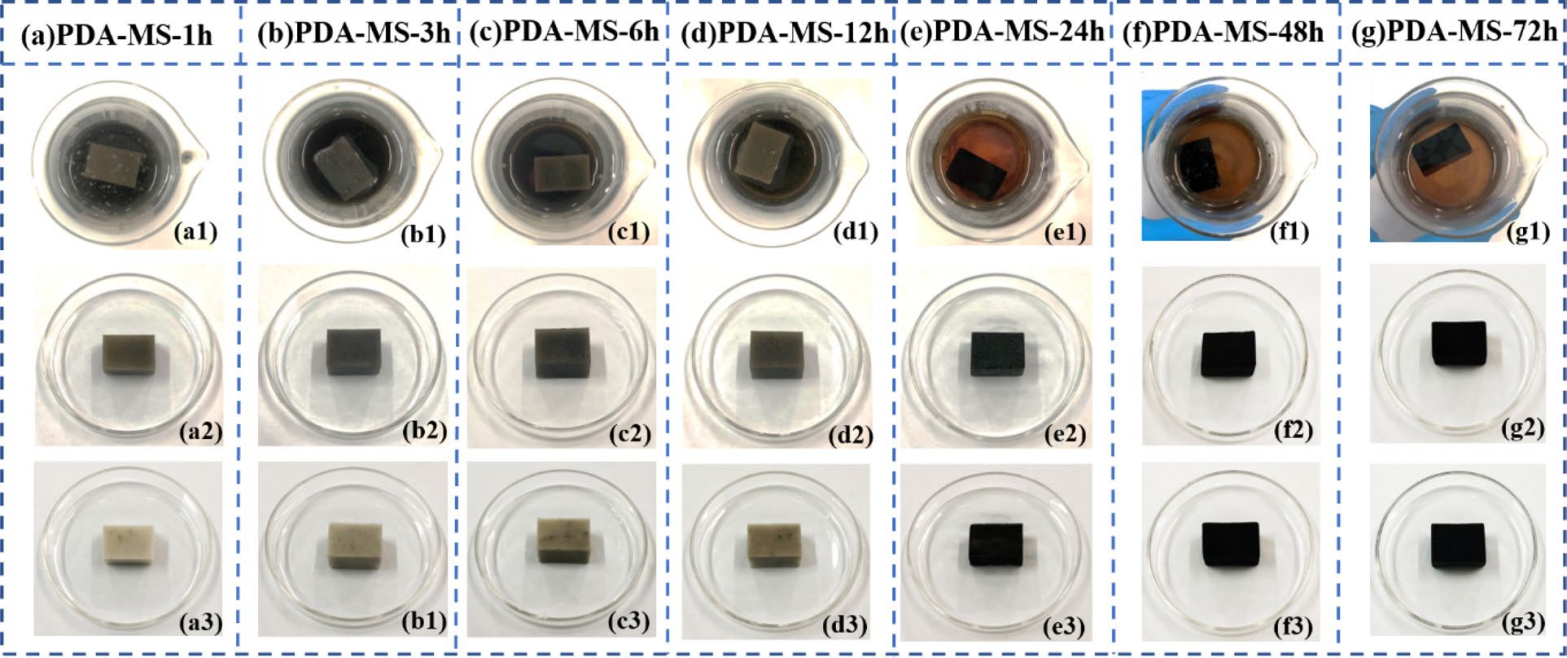
(**a1**–**g1**) the different MS dipped in PDA suspension after polymerized for 1–72 h; (**a2**–**g2**) Color of PDA-MS-1 h to PDA-MS-72 h after polymerization for 1 h-72 h; (**a3**–**g3**) Color of PDA-MS-1 h to PDA-MS-72 h after extruded the excess PDA solution.

The polymerization time of PDA effected the uniformity and quantity of PDA particles on the surface of MS. Furthermore, the uniformity and quantity of PDA particles could influence the secondary adhesion strength of PDA molecules and hydrophobic agent. As shown in Fig. 10a1–g1, the color of PDA suspension was black before polymerization for 12 h, and the color of the PDA suspension gradually changed from black to coffee after 24 h polymerization. This phenomenon suggested that the PDA particles in the solution self-polymerized and deposited on the skeleton surface of the MS after polymerization for 24 h. As illustrated in Fig. 10a2–g2, the PDA-MS before polymerization for 1–12 h possessed brown color, and however, the sponge after polymerization for 24–72 h basically possessed black appearance, which indicated that PDA particles polymerized quite quickly on the sponge skeleton, and after polymerization for 24 h the particles were able to cover the skeleton surface of the sponge. As described in Fig. 10a3–g3, after the PDA-MS squeezed out the excess solution, the color of the PDA-MS deepened and turned to dark brown with the increase of polymerization time, indicating that although PDA particles could quickly cover on the surface of the sponge skeleton, PDA could not be firmly deposited on the surface of the sponge skeleton in a short time (1–12 h). This might be attributed to that the PDA particles could not form a stable connection after self-aggregation on the surface of sponges. Before polymerization for 12 h, only a few PDA particles were firmly deposited on the surface of the sponge skeleton. With the increase of polymerization time, the PDA particles could self-polymerize continuously on the sponge surface, and the PDA molecules formed stable covalent and hydrogen bonds on the sponge skeleton surface. Therefore, after polymerization for 24 h, when the sponges was extruded excessive solution, it still could exhibite black color which was similar with the color of dopamine particles.

**Figure 11:**
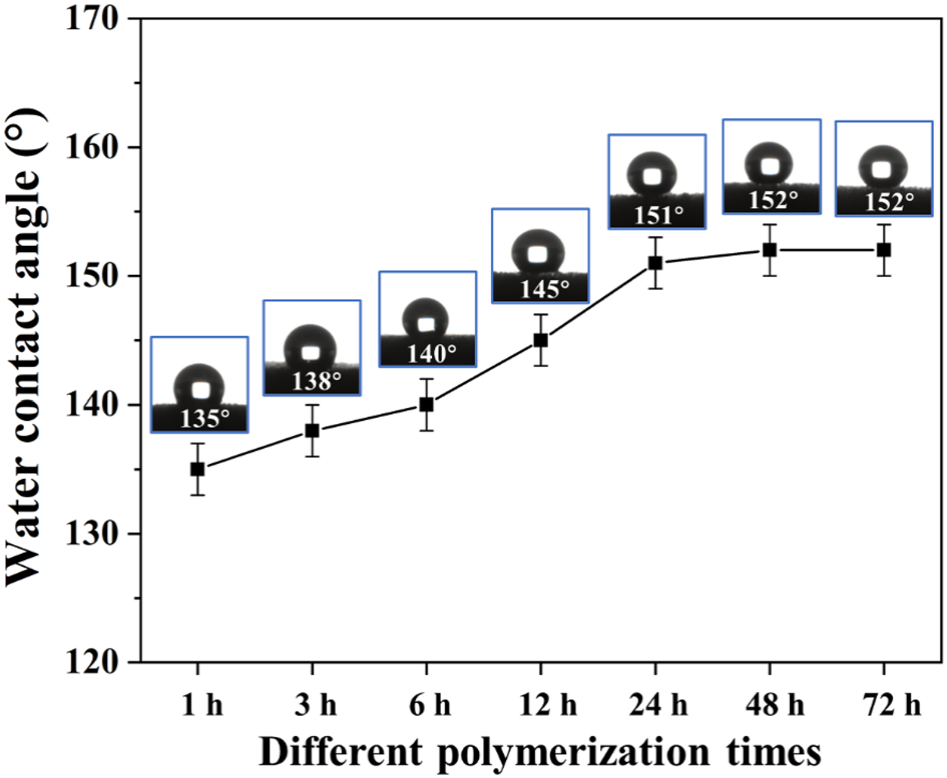
The WCAs of OTS-PDA-MS with different polymerization time in PDA suspension.

As can be seen from Fig. 11, with the increase of polymerization time of PDA-MS, the WCA of OTS-PDA-MS was increased after hydrophobic modification. However, the WCA had subtle variation from 151°to 152°after polymerization for 24–72 h, which was attributed to that the number of PDA particles on the surface of PDA-MS had tiny change after polymerization for 24–72 h, and the strength of secondary adhesion of PDA-MS was similar. Therefore, the WCA of OTAS-PDA-MS with different polymerization time was similar. This phenomenon indicated that the WCA of OTAS-PDA-MS was closely related to the polymerization times of PDA particles deposition on the surface of sponge. Therefore, it could be concluded that the hydrophobicity of the sponge was depend on the polymerization time and the amount of PDA particles deposited on the sponge skeleton. With the increase of polymerization time of PDA-MS, the number of PDA particles and the secondary adhesion of PDA-MS was enhanced, and morover, the hydrophobicity of OTS-PDA-MS was enhanced.

### Effect of different dosages of OTS

**Figure 12:**
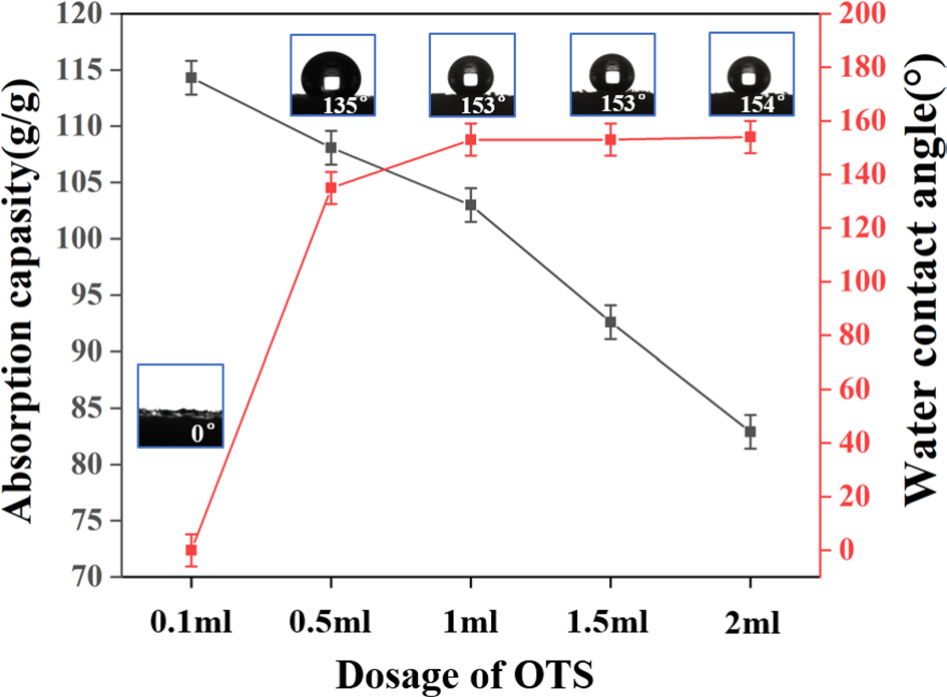
Absorption capacity and the WCAs of OTS-PDA-MS as a function of different dosages of OTS.

In the hydrophobic modification process of OTS-PDA-MS, the dosage of OTS is a crucial factor affecting the hydrophobicity and absorbability of OTS-PDA-MS, and before adding OTS, every sponge was sufficiently modified with PDA, which ensured that the PDA particles and PDA film covered the surface of the sponge completely. Therefore, the impact of PDA dosage on the absorption capacity has tiny inference. The volume ratio of OTS and ethanol was fixed at 1:40, and the dosages of OTS were 0.1 ml, 0.5 ml, 1 ml, 1.5 ml and 2 ml. Figure 12 shows the effect of OTS dosage on the absorption capacity of OTS-PDA-MS; it was apparent that with increasing OTS dosage from 0.1 ml to 2 ml, the absorption capacity of OTS-PDA-MS for lubricating oil decreased rapidly, and when the dosage of OTS was 2 ml, the absorption capacity of OTS-PDA-MS was 59.3 g/g. The WCA of OTS-PDA-MS showed slight differences when the dosages of OTS were 1 ml, 1.5 ml and 2 ml, and when the dosage of OTS was 0.1 ml, the WCA of OTS-PDA-MS was 0°. Therefore, a dosage of 0.1 ml only slightly affected the water repellency of OTS-PDA-MS, while the WCAs of OTS-PDA-MS reached 154° when the dosage of OTS was 2 ml. The dramatic wettability variation of OTS-PDA-MS as a function of the different dosages of OTS can be correlated with the loading of OTS on the surface of PDA-MS. As shown in Fig. 12, when the dosage of OTS exceeded 1 ml, the loading of OTS had little influence on the wettability of OTS-PDA-MS.

**Figure 13:**
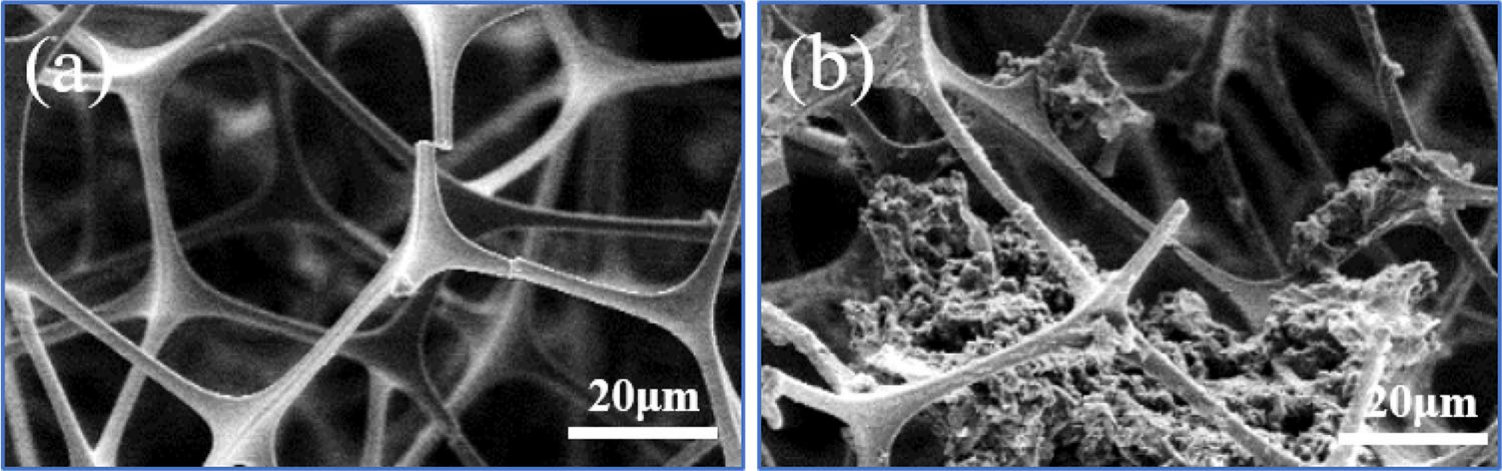
SEM images of OTS-PDA-MS for different dosages of OTS. (**a**) 0.1 ml of OTS; (**b**) 2 ml of OTS.

However, with increasing OTS dosage, the absorption capacity of OTS-PDA-MS sharply decreased, which was attributed to the high loading of OTS on the surface of PDA-MS blocking the channels of the sponge, resulting in a decrease in the oil absorption capacity of OTS-PDA-MS. As seen in Fig. 13a, b, with the increase in OTS dosage from 0.1 to 2 ml, the amount of OTS attached to the walls of the pore channels of the OTS-PDA-MS increased, which resulted in a reduction of the pore size and correspondingly decreased the oil absorption capacity of OTS-PDA-MS^53^. In conclusion, the hydrophobicity of the sponge was dependent on the loading of OTS, which also affected the oil absorption capacity of the sponge.

### Absorption capacity of OTS-PDA-MS for oils and organic solvents

In this study, OTS-PDA-MS showed a considerable absorption capacity for oils and organic solvents. As illustrated in Table 1, the absorption capacities of OTS-PDA-MS for oils and organic solvents reached 93.2–165.9 g/g. The reason that the absorption capacity of phenixin has reached 165.9 g/g is because the large density of phenixin.

**Table 1:** Absorption capacity of OTS-PDA-MS for oil/solvents with different viscosities and densities.

Oil/solvents	Viscosity	Density (cm^3^)	Absorption (g/g)
Acetone	0.3–0.4 mPa s	0.7845	104.48 ± 2.25
Methanol	0.55 mPa s	0.7918	102.84 ± 1.85
Butyl alcohol	2.948 mPa s	0.8098	93.92 ± 0.87
Phenixin	0.965 mPa s	1.595	166.42 ± 1.53
Lubricate oil	242.4 cSt 25 °C	0.8510	94.02 ± 0.98
Pump oil	243 cSt 25 °C	0.879	97.08 ± 1.14
Diesel oil	0.05 Pa s 25 °C	0.72	96.66 ± 2.00
Sunflower oil	0.2 Pa s 25 °C	0.91	104.92 ± 1.36

**Figure 14:**
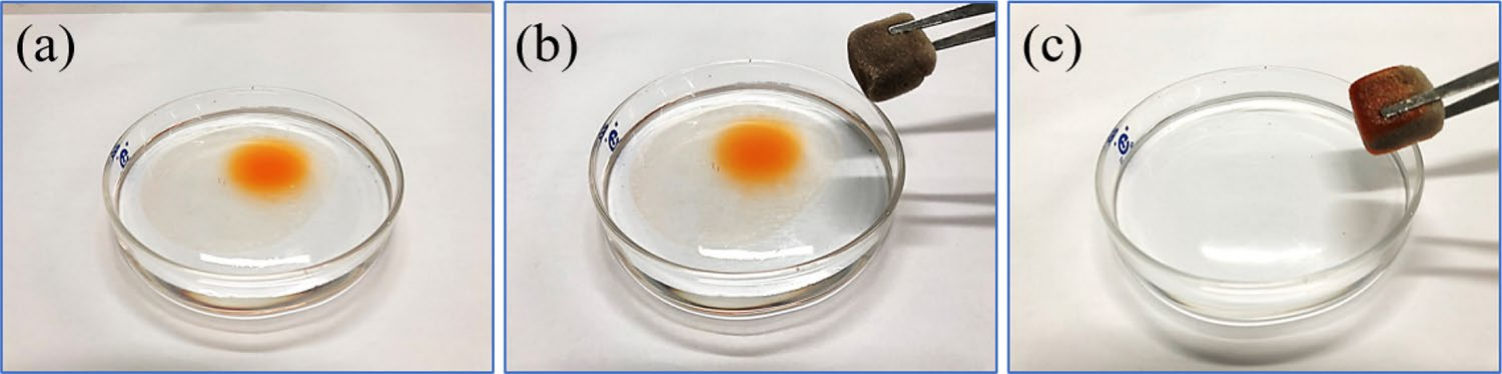
Oils dyed with Sudan III were absorbed by OTS-PDA-MS on the surface of water.

As illustrated in Fig. 14a–c, when a piece of OTS-PDA-MS contacted the lubricating oil layer floating on the surface of the water, the oil layer continuously decreased in thickness and was quickly absorbed by the sponge in a few seconds. Additionally, it can be seen in Video S3 that when the OTS-PDA-MS was swept over the lubricating oils on the surface of water, only clean water was left in the dish, and red oil attached tightly to the sponge. In contrast, the raw MS absorbed the lubricating oil and part of the water simultaneously, as shown in Video S4, which can be attributed to the amphiphilicity of the raw MS.

**Figure 15:**
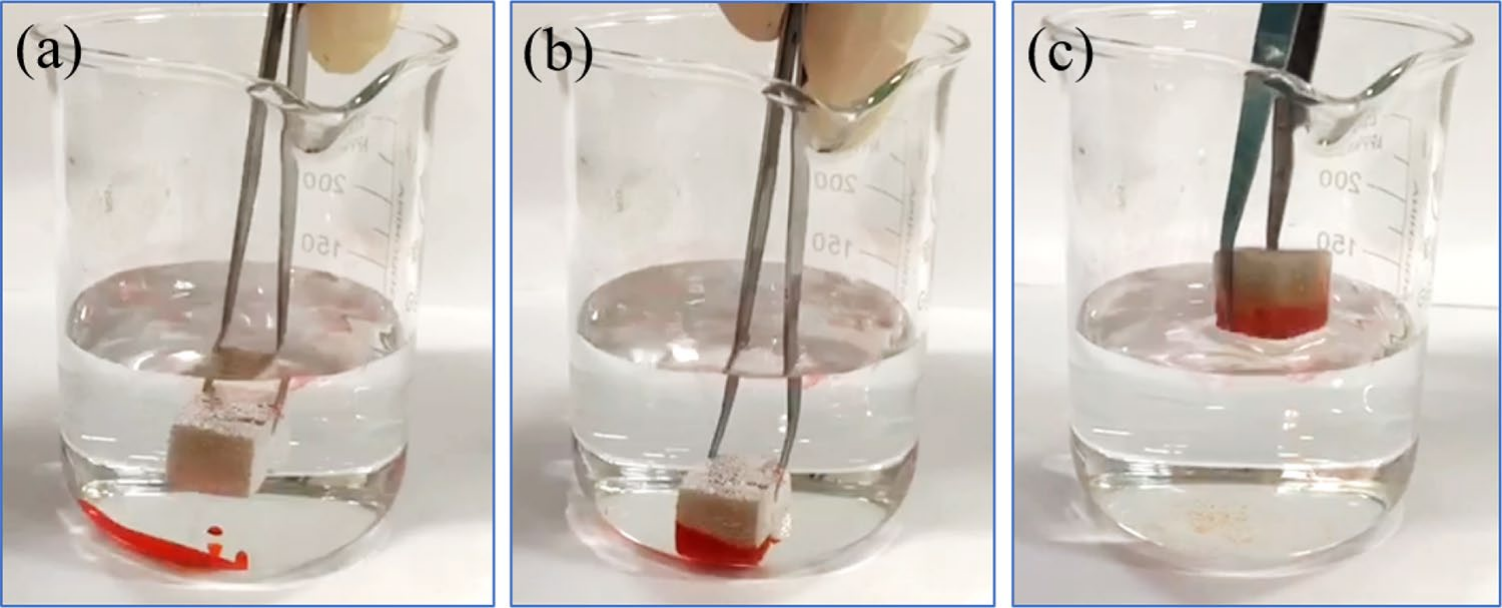
Tetrachloromethane dyed with Sudan III was absorbed by OTS-PDA-MS when injected at the bottom of the beaker.

As shown in Fig. 15a–c, tetrachloromethane (dyed with Sudan III) injected at the bottom of the beaker was removed immediately when OTS-PDA-MS was brought in contact with tetrachloromethane. As displayed by Video S5, tetrachloromethane was directly absorbed when it contacted OTS-PDA-MS, and at the same time, the air trapped in the interior pores of OTS-PDA-MS was replaced by tetrachloromethane, and air bubbles appeared in the water. The main reason for this phenomenon is that when tetrachloromethane was absorbed into the inner pores of OTS-PDA-MS, the air in the pores of the sponge was forced out, which resulted in the formation of bubbles. Moreover, the absorbed oils could easily be recovered through a simple physical process of centrifugation, which is an eco-friendly and time-efficient process.

The study results confirmed that OTS-PDA-MS exhibits excellent selectivity for the absorption of oil on the surface of water or under water, and therefore, the OTS-PDA-MS developed in this study shows great potential for practical applications in oil spill treatment.

### Recyclability

**Figure 16:**
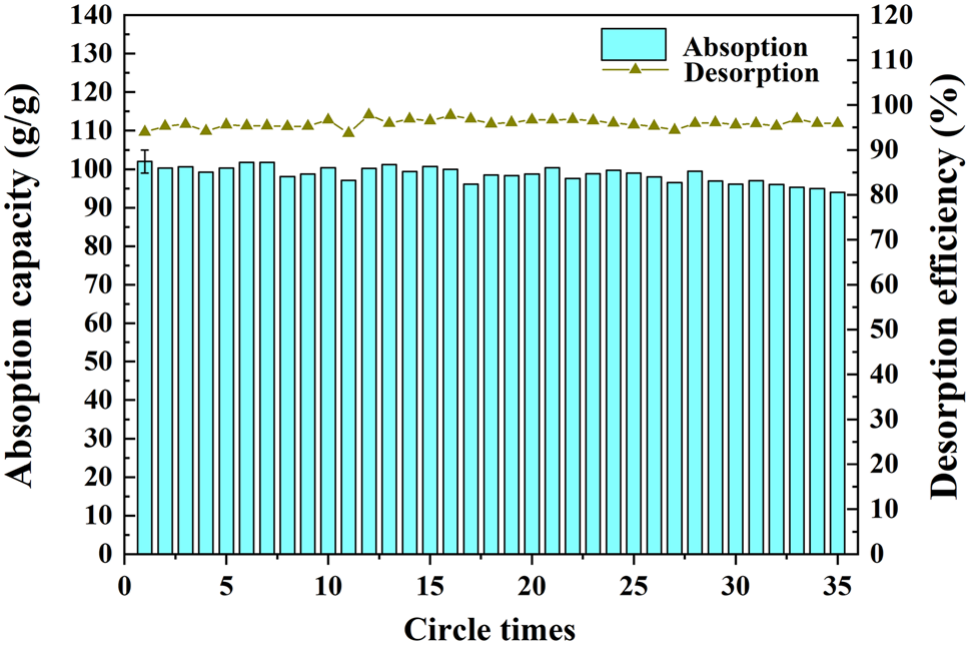
Absorption–desorption recyclability of OTS-PDA-MS for sunflower oils.

Recyclability is a crucial parameter for practical oil clean-up applications when absorbing oil spills. As illustrated in Fig. 16, OTS-PDA-MS reached at least 94% oil desorption efficiency, while the absorption capacity of OTS-PDA-MS remained at approximately 94 g/g even after 35 cycles. The small decrease in the oil absorption capacity of OTS-PDA-MS after the absorption–desorption recycling tests was mainly attributed to the following reasons: First, in the desorption process, some residual sunflower oils were retained in the internal pores of OTS-PDA-MS, leading to a decrease in the oil absorption capacity of OTS-PDA-MS. Second, in the absorption–desorption process, the hydrophobic layer on the surface of OTS-PDA-MS and the structure of OTS-PDA-MS were destroyed, thereby causing a decrease in the hydrophobicity and oil retention capacity of OTS-PDA-MS.

**Figure 17:**
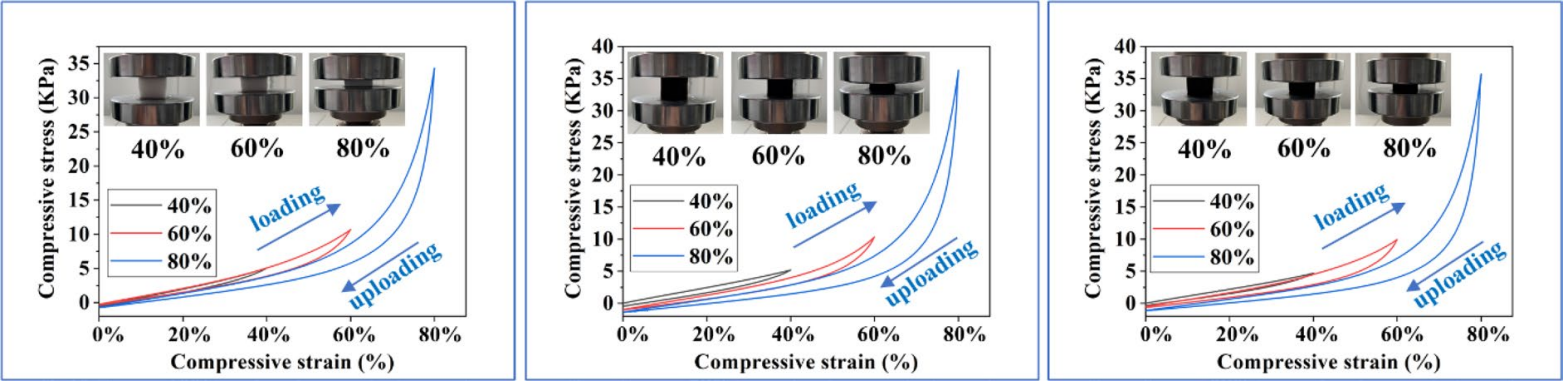
The stress–strain curves of the superhydrophobic MS at different strain of 40%, 60%, and 80%, respectively. (**a**) MS; (**b**) OTS-PDA-MS (**c**) OTS-PDA-MS after reused. And the Inset photographs of sponges during the compression process.

Mechanical stability of the materials is critical to their practical application. The compressibility of MS, OTS-PDA-MS and reused OTS-PDA-MS is displayed in Fig. 17, respectively. The hysteresis loop of stress–strain curves was attributed to the 3D porous structure of MS and the stress–strain curves of MS, OTS-PDA-MS and reused OTS-PDA-MS were nearly the same. Moreover, the OTS-PDA-MS and reused OTS-PDA-MS can could easily recover from 40, 60 to 80% of compression strain. The substrate MS exhibits excellent flexibility, meanwhile the OTS and PDA coatings did not affect the inherited elasticity of MS. Furthermore, the OTS-PDA-MS after reused also retain the considerable resilience.

**Figure 18:**
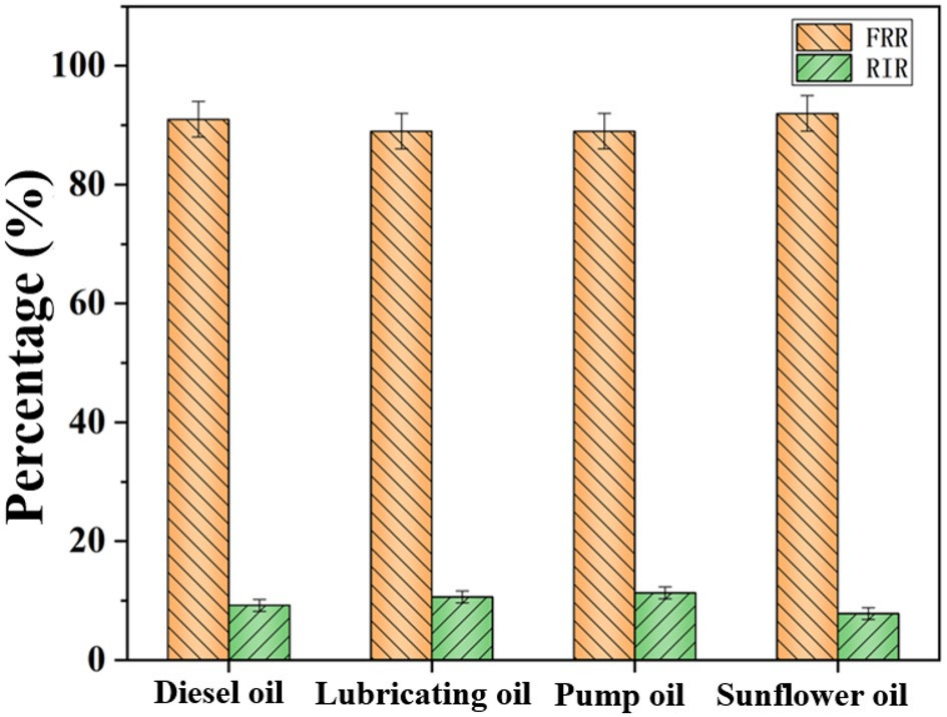
Flux recovery ratio (FRR), and irreversible fouling ratio (RIR) of OTS-PDA-MS for oil absorption with various oil.

For purpose of further test the anti-oil fouling property of sponges, different kinds of oil were absorbed by OTS-PDA-MS and then the OTS-PDA-MS was desorption by centrifugation. It was observed from the analysis of oil resistance (Fig. 18) that the OTS-PDA-MS could absorb different kinds of oil that showed higher FRR and lower RIR values. These results showed that the oil stored on the skeleton of OTS-PDA-MS could be removed by centrifugation, which could significantly inhibit the irreversible scaling of oil pollution on the skeleton of OTS-PDA-MS.

**Figure 19:**
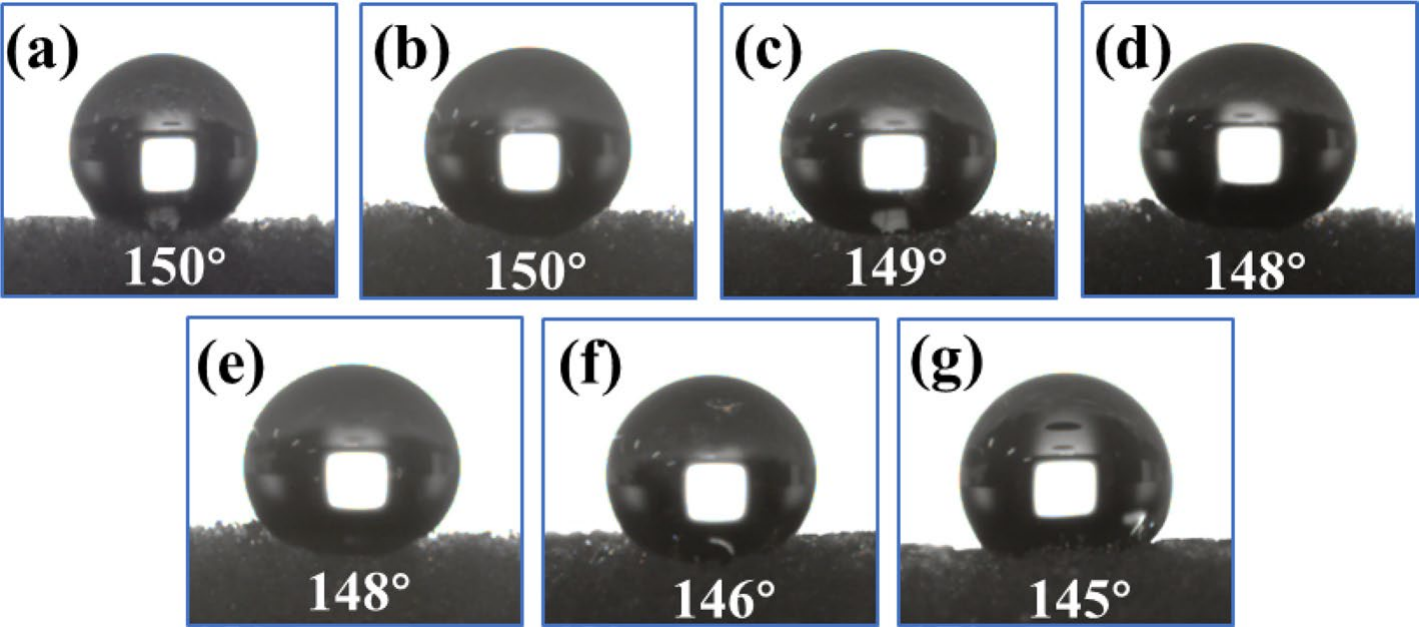
WCA of the sponge after every five absorption–desorption cycles: (**a**) 5th cycle; (**b**) 10th cycle; (**c**) 15th cycle; (**d**) 20th cycle; (**e**) 25th cycle; (**f**) 30th cycle; (**g**) 35th cycle.

As illustrated in Fig. 19a–g, the WCA of OTS-PDA-MS decreased from 150° to 145° during the absorption–desorption process after 35 cycles, which indicated that the hydrophobic layer on the surface of OTS-PDA-MS deteriorated gradually. After 35 cycles, although OTS-PDA-MS could not achieve super-hydrophobicity, it still retained its prominent hydrophobicity. Therefore, the study results showed that OTS-PDA-MS not only had excellent absorbability for oil or organic solvents but also exhibited good desorption and reuse ability.

The absorption capacity and hydrophobicity of OTS-PDA-MS were compared with other absorbent materials based on sponges previously reported, and the results are listed in Table 2. Compared to the other excellent sponge materials, OTS-PDA-MS not only possessed a high absorption capacity for phenixin but also exhibited a remarkable water contact angle before and after absorption–desorption recycling by the simple centrifugation method. Moreover, dopamine biomimetic bonding has the advantages of being facile, environmentally friendly and cost-effective. Therefore, the superhydrophobic sponge developed in this study has great potential in practical applications for oil absorption.

**Table 2:** Comparison of various superhydrophobic sponges.

Samples	Solvent/oil	Sorption	Linked materials	Hydrophobic materials	Environmental impact	Water contact angle	WCA after recycle	References
**Polyurethane**								
Sponge	Rapeseed oil	43	Attapulgite	OTS	Little	160°	/	^54^
MS	Chloroform	53	SiO_2_	OTS and Polyfluorowax	Medium	150°	/	^13^
MS	Motor oil	40.5	GPTS/APTES	OTS	Medium	151.39°	142.13°	^19^
MS	Chloroform	165	Graphene nanosheets	PDMS	Little	162°	/	^26^
**Polyurethane**								
Sponge	Silicone oil	43.3	SiO_2_	Poly(vinylidene fluoride)	Medium	154.3°	/	^23^
CNT sponge	Chloroform	176	/	1,2-dichlorobenzene as the carbon source	Little	/	/	^55^
Poly(melamine–formaldehyde) sponge	Chloroform	145	/	Lighting the toluene on the sponge surface	Medium	149°	/	^56^
Melamine sponge	Phenixin	165.9	PDA	OTS	Little	153°	145°	This work

The “/” represents the data were not given in the references.

### Dopamine biomimetic bonding used for the hydrophobic modification of other types of sponges

In addition to the MS, the polyethylene sponge and polyurethane sponge were also modified by OTS based on PDA biomimetic bonding to prepare OTS-PDA-PES and OTS-PDA-PUS, and their density, porosity, oil absorption, and WCA are shown in Table 3. The WCA and the oil absorption amounts of the sponges with different porosities and densities varied greatly, even when prepared with the same modification method. OTS-PDA-PUS-3 with high porosity absorbed pump oil with the lowest absorption of 19.4 g/g. OTS-PDA-PUS-1 with the lowest porosity absorbed pump oil up to 29.7 g/g. Moreover, comparing the two polyethylene sponges (PES), the absorption capacity of OTS-PDA-PES-2 with higher porosity for pump oil was 56.9 g/g, which was higher than that of OTS-PDA-PES-1. Moreover, the MS with the highest porosity and low density possessed the largest absorption capacity for pump oil. Therefore, the increase in the porosity of the sponge was beneficial for enhancing its absorption capacity.

**Table 3:** Comparison of different types of sponges . modified by PDA and OTS.

	OTS-PDA-MS	OTS-PDA-PES-1	OTS-PDA-PES-2	OTS-PDA-PUS-1	OTS-PDA-PUS-2	OTS-PDA-PUS-3
Lubricate oil absorption	96.7 g/g	41.1 g/g	56.9 g/g	32.3 g/g	25.4 g/g	21.3 g/g
Phenixin absorption	169.5 g/g	73.5 g/g	96.3 g/g	55.7 g/g	44.2 g/g	41.1 g/g
WCA	153°	143°	145°	143°	142°	141°
Density	0.00845 g/cm^3^	0.0206 g/cm^3^	0.0114 g/cm^3^	0.0194 g/cm^3^	0.0298 g/cm^3^	0.0431 g/cm^3^
Porosity	99.46%	98.3%	98.75%	98.1%	97.1%	96.8%

Additionally, the WCA of OTS-PDA-MS using the MS as the raw material was higher than that of OTS-PDA-PUS and OTS-PDA-PES using polyurethane sponge and polyethylene sponge as the raw materials, which might be caused by hydroxy and amino groups in PDA interacting with methyl groups in polyethylene and carbamate groups in polyurethane to form unstable connections, leading to the incomplete modification of PDA and OTS on the sponge surfaces. However, according to the study results, all of the hydrophobic sponges prepared in this paper possessed favorable oil absorption capacity; therefore, the dopamine biomimetic bonding superhydrophobic modification method developed in this study can be used for different types of sponges.

## Conclusion

A novel method based on PDA biomimetic bonding was developed for the fabrication of superhydrophobic sponges for oil spill treatment. The as-prepared superhydrophobic sponge of OTS-PDA-MS satisfied the basic demands of oil removal materials: excellent hydrophobic water contact angle of 153°, high selective absorption capacity of 165.9 g/g, and more importantly, excellent reusability, which remained at approximately 92.1% of its initial absorption capacity after 35 cycles. Additionally, the proposed hydrophobic modification method can substantially enhance the hydrophobicity of almost all types of sponge materials, including sponge wastes. Because of the cost-effective materials, feasible fabrication technique and excellent performance, we envision that this research could extend the application range of PDA and sponges for oil removal.

## Supplementary Information

Supplementary Video 1.

Supplementary Video 2.

Supplementary Video 3.

Supplementary Video 4.

Supplementary Video 5.
